# Chloride load and serum chloride in critically ill adults: a randomization-anchored analysis of the BaSICS trial

**DOI:** 10.1016/j.aicoj.2026.100191

**Published:** 2026-10-22

**Authors:** Fernando G. Zampieri, Israel S. Maia, Flavia R. Machado, Alexandre B. Cavalcanti

**Affiliations:** a Department of Critical Care Medicine, University of Alberta, Edmonton, Alberta, Canada; b Research Institute, HCor–Hospital do Coração, São Paulo, SP, Brazil; c Intensive Care Department, Hospital São Paulo, Escola Paulista de Medicina, Universidade Federal de São Paulo, São Paulo, Brazil

**Keywords:** Balanced crystalloids, Chloride, Critical illness, Fluid therapy, Saline solution

## Abstract

**Background:**

0.9% saline solution has a supraphysiological chloride concentration and has been associated observationally with acute kidney injury and mortality. How randomized fluid type changes serum chloride early in critical illness, and what determines this change, remain uncertain. Focusing on the first three days after randomization, we estimated the randomized saline-versus-Plasma-Lyte difference in serum chloride and its variation by renal function, the observational association with study-saline volume, and the sources of variation in serum chloride.

**Methods:**

This secondary analysis of BaSICS (75 Brazilian ICUs; Plasma-Lyte 148 vs 0.9% saline) included participants with baseline serum chloride and at least one measurement on days 1–3. A Bayesian hierarchical longitudinal model estimated the treatment effect over time and its variation by baseline estimated glomerular filtration rate (eGFR). Group-level analyses used day 2, which balanced accumulated exposure against attrition of measurements. We normalized randomized between-group differences, estimated an inverse-probability-weighted within-saline volume association, and partitioned modeled variation in serum chloride.

**Results:**

Among 3,482 patients (33% of the trial population), serum chloride rose 2.15 mmol/L (95% confidence interval, 1.76–2.53) more with saline than with Plasma-Lyte by day 2, when saline recipients had received on average 145.7 mmol more study-fluid chloride, corresponding to 1.47 mmol/L per 100 mmol of additional chloride (1.17–1.81). Within saline recipients, the weighted association was +0.31 mmol/L per 100 mmol (0.22 to 0.44). The day-2 between-group difference decreased by 0.29 mmol/L (95% credible interval, 0.12 to 0.46) per 15 mL/min/1.73 m^2^ higher baseline eGFR (+3.0 mmol/L at 30 vs +1.8 mmol/L at 90 mL/min/1.73 m^2^). Of modeled variation in serum chloride, the fluid component accounted for 0.7% (0.4–1.0; 1.8% with randomized arm included); measured baseline characteristics, almost entirely baseline serum chloride, for 48.1%; persistent patient-specific differences not explained by measured characteristics for 24.5%; and day-to-day within-patient fluctuation for 26.9%.

**Conclusions:**

Over the first three ICU days, randomization to saline increased serum chloride relative to Plasma-Lyte, and the between-group difference was larger at lower baseline eGFR. Most modeled variation in serum chloride reflected each patient's baseline chloride and persistent patient-specific differences that measured characteristics did not explain; the fluid component was small.

**Trial registration:**

ClinicalTrials.gov, NCT02875873.

## Introduction

0.9% sodium chloride solution (saline) is widely used in clinical practice. Its chloride concentration of 154 mmol/L substantially exceeds the normal plasma chloride concentration. Hyperchloremia has been associated with higher hospital mortality in critically ill patients with sepsis [1]. Multiple randomized trials have compared balanced crystalloids with saline to determine whether fluid choice improves clinical outcomes [[2], [3], [4]]. Mortality findings have generally been neutral. A recent individual participant data meta-analysis found a high posterior probability that balanced crystalloids reduce in-hospital mortality, with moderate certainty of evidence and a small estimated absolute effect [5,6]. Across trials, balanced crystalloids have consistently produced serum chloride concentrations approximately 1−2 mmol/L lower than saline. This biochemical separation alongside small or uncertain mortality effects raises several possibilities: serum chloride may be a weak mediator; benefits may arise through other mechanisms; or renal effects may be too small to alter survival.

How administered chloride is reflected in serum chloride concentrations in critically ill patients remains poorly characterized. Serum chloride is homeostatically regulated. The most direct experimental evidence comes from a randomized crossover study in 12 healthy male volunteers in which a 2-L infusion of 0.9% saline over 1 h reduced renal artery blood-flow velocity and renal cortical tissue perfusion from baseline, whereas Plasma-Lyte 148 did not [7]. Critical illness commonly impairs renal excretory capacity and endothelial function and alters fluid distribution, limiting direct applicability of healthy-volunteer findings.

Randomization supports estimation of the effect of assigned fluid type on serum chloride. Saline and Plasma-Lyte differ in multiple constituents, making assigned fluid type the randomized estimand. In this secondary analysis of the BaSICS (Balanced Solutions in Intensive Care Study) trial [3], which randomized more than 10,000 critically ill adults to receive 0.9% saline or Plasma-Lyte 148, we focused on the first three days after randomization, when study-fluid exposure was concentrated and serum chloride was recorded daily. We therefore addressed the short-term biochemical response rather than serum chloride over the entire ICU stay. We aimed to quantify the randomized between-group difference in serum chloride; estimate, among saline recipients, the observational association between study-fluid volume and change in serum chloride; and partition modeled variation in serum chloride. The partition separated administered fluid and study day from three patient sources: measured baseline characteristics, chiefly each patient's starting serum chloride; persistent patient-specific differences, meaning a patient's tendency to run consistently higher or lower than predicted from measured characteristics; and day-to-day fluctuation within the same patient.

## Methods

### Design

We performed a secondary analysis of de-identified data from the BaSICS trial, a double-blind, 2 × 2 factorial randomized clinical trial conducted in 75 intensive care units (ICUs) in Brazil between May 2017 and March 2020 (ClinicalTrials.gov, NCT02875873) [3]. The parent trial obtained written informed consent according to its protocol. Additional participant consent was not required for this secondary analysis of de-identified data.

### Participants

BaSICS enrolled adults admitted to an ICU who required at least one fluid expansion at the treating physician's discretion, were not expected to leave the ICU the following day, and met at least one prespecified risk criterion for acute kidney injury: age older than 65 years; hypotension (mean arterial pressure <65 mmHg, systolic blood pressure <90 mmHg, or vasopressor use); sepsis; invasive or noninvasive ventilatory support, including high-flow nasal cannula, for at least 12 h; early kidney dysfunction (urine output <0.5 mL/kg/h for at least 3 h or serum creatinine >1.2 mg/dL in women or >1.4 mg/dL in men); or cirrhosis or acute liver failure. Exclusion criteria were current or expected kidney replacement therapy within 6 h, severe dysnatremia (serum sodium ≤120 or ≥160 mmol/L), expected death within 24 h, suspected or confirmed brain death, exclusive palliative or comfort care, or previous enrollment. Hyperkalemia (serum potassium >5.5 mEq/L) remained an exclusion criterion until its removal after the second interim analysis [3].

### Study intervention

Participants were randomized independently to fluid type (Plasma-Lyte 148 or 0.9% saline) and fluid-challenge infusion rate in a 2 × 2 factorial design; the present analysis focused on the fluid-type comparison. The study fluids were supplied in indistinguishable 500-mL bags. Sites were asked to use the allocated fluid for all fluid challenges, maintenance fluids, and compatible drug infusions greater than 100 mL during the ICU stay, for up to 90 days. The indication, timing, and volume of fluid administration remained at the treating clinician's discretion, and non-study fluids could be used when clinically indicated [3].

### Analytic cohort

The present analysis included participants with a baseline serum chloride measurement and at least one postbaseline serum chloride measurement on days 1–3 after randomization. Serum chloride measurement was not mandated by the trial protocol; the treating team measured it according to clinical indication and local practice.

### Variables

The primary exposure was cumulative post-randomization study-fluid chloride load (mmol), calculated daily as the recorded study-fluid volume multiplied by the assigned fluid's chloride concentration (154 mmol/L for saline and 98 mmol/L for Plasma-Lyte 148), divided by 1,000, and summed from day 1 through the corresponding study day. One implausible day-2 entry of 65,000 mL, recorded for both study and total fluid, was treated as a data-entry error and corrected to 5,000 mL before exposure construction. Pre-enrollment and non-study-fluid chloride were excluded from the primary exposure. The intermediate variable was change in serum chloride from baseline. The trial recorded serum chloride at baseline and on days 1, 2, 3, and 7; this analysis used days 1–3, when study-fluid exposure was concentrated and most measurements were available. Day-7 values were available for only 794 (23%) participants, all still in the ICU, and were not analyzed. No missing values were imputed. The longitudinal model used all available postbaseline serum chloride measurements among participants with the required baseline variables; other models used complete cases for their required variables. We calculated eGFR at randomization using the 2021 Chronic Kidney Disease Epidemiology Collaboration (CKD-EPI) equation and serum creatinine recorded in mg/dL, measured after hospital admission and closest to randomization (day 0). Baseline covariates used for weighting or effect-modification analyses, all recorded at randomization, were age, sex, Sequential Organ Failure Assessment (SOFA) and Acute Physiology and Chronic Health Evaluation (APACHE) II scores, admission type, baseline serum chloride, baseline eGFR, baseline serum sodium, measured body weight (as reported by the enrolling site), mean arterial pressure, and vasopressor use. They were chosen on clinical grounds as pre-treatment determinants of illness severity, hemodynamic instability, body size, renal function, and electrolyte status, which may influence both the volume of fluid prescribed and serum chloride; because they were recorded at randomization, they could not be affected by the assigned fluid.

### Statistical analysis

Day 2 served as the reference day for the descriptive and group-level analyses. Among participants with paired measurements, the between-group difference in cumulative study-fluid chloride load was 100.0 mmol by day 1 (3,205 participants), 145.7 mmol by day 2 (2,721 participants), and 206.8 mmol by day 3 (1,982 participants). Day 1 therefore offered the smallest exposure contrast, whereas by day 3 a further 27% of participants had no measurement as patients left the ICU or died. Day 2 balanced accumulated exposure against sample size and attrition; the longitudinal model used all three days. We first descriptively summarized mean change in serum chloride from baseline, with 95% confidence intervals, by randomized group over days 1–3 and, at day 2, by randomized group and baseline APACHE II quartile among participants with paired measurements. These analyses were unadjusted. We then performed three principal analyses: a randomized longitudinal fluid-type comparison with eGFR effect modification; a group-level normalization of randomized between-group differences and an observational within-saline volume association; and a partition of modeled variation in serum chloride.

First, we estimated how the randomized difference in serum chloride between saline and Plasma-Lyte changed over days 1–3 and whether that difference varied by baseline renal function. We fitted a Bayesian hierarchical longitudinal model to all available serum chloride measurements; each patient contributed one to three observations. The linear predictor was:

Chloride ∼ arm × day × eGFRc + baseline chloride + (1 + day | patient)

In this model, arm indicated randomized fluid (Plasma-Lyte 148 as the reference and 0.9% saline as the comparison); day was entered as a three-level categorical variable in the fixed effects, allowing day-specific treatment effects; eGFRc was baseline eGFR centered at 90 mL/min/1.73 m^2^ and scaled per 15 mL/min/1.73 m^2^ [eGFRc = (eGFR − 90)/15]; and baseline chloride was centered at 106 mmol/L. Numeric study day was used for the patient-specific random slope. From this model, we derived, for each study day, how much the randomized saline-versus-Plasma-Lyte difference changed with each 15 mL/min/1.73 m² higher baseline eGFR. These contrasts tested effect modification by eGFR within the randomized fluid assignment; fluid volume remained clinician-determined. Adjustment for baseline chloride estimated the treatment effect conditional on the initial value, and correlated patient-specific random intercepts and slopes accounted for repeated measurements. The model included 7,892 measurements from 3,474 participants; computed eGFR values outside 5-200 mL/min/1.73 m² were excluded from this model. We placed weakly informative normal (0, 10) priors on all fixed-effect coefficients and sampled the Gaussian likelihood using four chains of 4,000 iterations. We estimated saline-versus-Plasma-Lyte contrasts by day and eGFR from the posterior expectation of the linear predictor at the centered baseline and reported posterior medians with 95% credible intervals. We assessed robustness with frequentist linear mixed models of the same structure (lme4, maximum likelihood), fitted among patients measured on all three days and with standardized baseline creatinine replacing eGFR.

Second, we calculated a group-level ratio by dividing the randomized saline-minus-Plasma-Lyte difference in mean day-2 serum chloride change by the corresponding difference in post-randomization study-fluid chloride load and scaling the result per 100 mmol. A stratified participant bootstrap provided the 95% confidence interval. Among complete-case saline recipients, we also estimated unweighted and inverse-probability-weighted associations between study-fluid chloride load and day-2 serum chloride change. Because saline concentration was fixed at 154 mmol/L, greater load represented greater clinician-determined saline volume. Stabilized generalized-propensity-score weights included baseline creatinine, body weight, SOFA score, mean arterial pressure, age, and baseline chloride; weight capping, bootstrap procedures, and balance diagnostics are detailed in Additional file 1. We also calculated an apparent chloride distribution volume from the randomized between-group differences.

Third, we asked how much of the statistical variation in observed serum chloride levels was associated with administered fluid, study day, and patient factors. The outcome was the observed serum chloride value on days 1–3. We used a Bayesian hierarchical model with cumulative study-fluid chloride load, cumulative total fluid volume, categorical study day, baseline chloride, age, baseline creatinine, baseline SOFA score, and a patient-specific random intercept. We partitioned modeled total variation into six components. The fluid component comprised study-fluid chloride load and total fluid volume. Measured baseline characteristics comprised baseline serum chloride, age, baseline creatinine, and baseline SOFA score. Persistent between-patient differences, estimated by the patient-specific random intercept, captured how far each patient's chloride values remained consistently above or below the values predicted from fluid, study day, and measured characteristics. Within-patient residual variation captured day-to-day fluctuation of each patient's values around that patient's own level. Because the fluid, study-day, and baseline-characteristic predictors are correlated, part of their explained variation is shared and cannot be assigned to any single block; this shared part is reported as a covariance component, which can be negative. We report posterior medians with 95% credible intervals. Detailed model specifications, variance-partition equations, sensitivity results, and sampling diagnostics are provided in Additional file 1.

### Sensitivity analyses

To assess sensitivity to non-study fluid across plausible chloride concentrations, we added the observed mean between-group difference in total non-study-fluid volume priced at either 98 or 154 mmol/L to the day-2 study-fluid load gap. We also repeated the variance partition among measurements with documented absence of kidney replacement therapy through the measurement day, using change in serum chloride from baseline as the outcome, and with randomized arm added to the fluid component. In an exploratory analysis, we related the estimated patient-specific intercepts to baseline variables not included in the variance model (serum sodium, arterial pH, mean arterial pressure, body weight, pre-enrollment saline volume, APACHE II score, vasopressor use, sex, admission type, randomized arm, and study site) using linear regression.

Analyses used R 4.6 (brms/Stan, lme4) with fixed random seeds; analysis code is openly available (see Availability of data and material).

## Results

### Cohort

Of 10,520 trial participants, 3,482 (33%) had baseline serum chloride and at least one measurement on days 1–3 and constituted the analytic cohort. Of these, 2,721 had paired baseline and day-2 measurements (1,357 saline and 1,364 Plasma-Lyte). Mean SOFA score was slightly higher in the analytic cohort than among remaining trial participants (5.3 vs 4.4). Randomized allocation was similar (49.9% vs 50.5% saline), and baseline characteristics were similar between groups. Median post-randomization study-fluid chloride load on days 1–3 was 390 [interquartile range, 231–644] mmol with saline and 245 [147–400] mmol with Plasma-Lyte (Table 1).

**Table 1:** Baseline characteristics and exposure, by randomized arm (analytic cohort: baseline plus ≥1 follow-up serum chloride).

	0.9% Saline (n = 1,736)	Plasma-Lyte (n = 1,746)
Age, y	62 [49–71]	61 [48–71]
Female, No. (%)	743 (43)	754 (43)
Measured body weight, kg	70 [60–80]	70 [61–81]
SOFA score	5 [3–7]	5 [3–7]
APACHE II score	12 [9–18]	12 [8–17]
Admission — elective / non-sepsis / sepsis, No.	930 / 555 / 250	932 / 551 / 263
Mean arterial pressure, mmHg	74 [65–87]	74 [65–88]
Vasopressor use, No. (%)	781 (45)	758 (43)
Baseline chloride, mmol/L	105 [103–109]	106 [102–109]
Baseline eGFR (CKD-EPI 2021), mL/min/1.73 m²	74 [52–98]	77 [53–99]
Creatinine at randomization, μmol/L	89 [71–121]	88 [71–118]
Baseline sodium, mmol/L	138 [135–141]	138 [135–141]
Study fluid, days 1–3, mL	2,532 [1,500–4,180]	2,500 [1,500–4,078]
Total fluid, days 1–3, mL	3,854 [2,000–5,922]	3,658 [2,000–5,950]
Study-fluid chloride load, days 1–3, mmol^a^	390 [231–644]	245 [147–400]

Data are median [interquartile range] unless otherwise noted and use available observations. Missingness was no more than 3 participants per arm for any variable except measured body weight (55 saline; 57 Plasma-Lyte); admission type was missing for 1 saline recipient. eGFR = estimated glomerular filtration rate; APACHE = Acute Physiology and Chronic Health Evaluation; SOFA = Sequential Organ Failure Assessment.

a Study-fluid chloride load was calculated for post-randomization days 1–3 from recorded study-fluid volume (after correction of one data-entry error) multiplied by the assigned fluid concentration (154 mmol/L for saline; 98 mmol/L for Plasma-Lyte); pre-enrollment and non-study-fluid chloride were excluded.

### Severity and chloride rise

Post-randomization study-fluid chloride load through day 2 varied across baseline APACHE II quartiles (saline-group medians, 308−385 mmol; Fig. 1). Within the saline group, the mean day-2 increase in serum chloride rose from +1.1 mmol/L (95% confidence interval, 0.7–1.5) in the lowest quartile to +2.6 mmol/L (2.0–3.2) in the highest; with Plasma-Lyte, mean change ranged from −0.6 to −0.2 mmol/L across quartiles, with all 95% confidence intervals within −1.1 to +0.4 mmol/L.

**Fig. 1:**
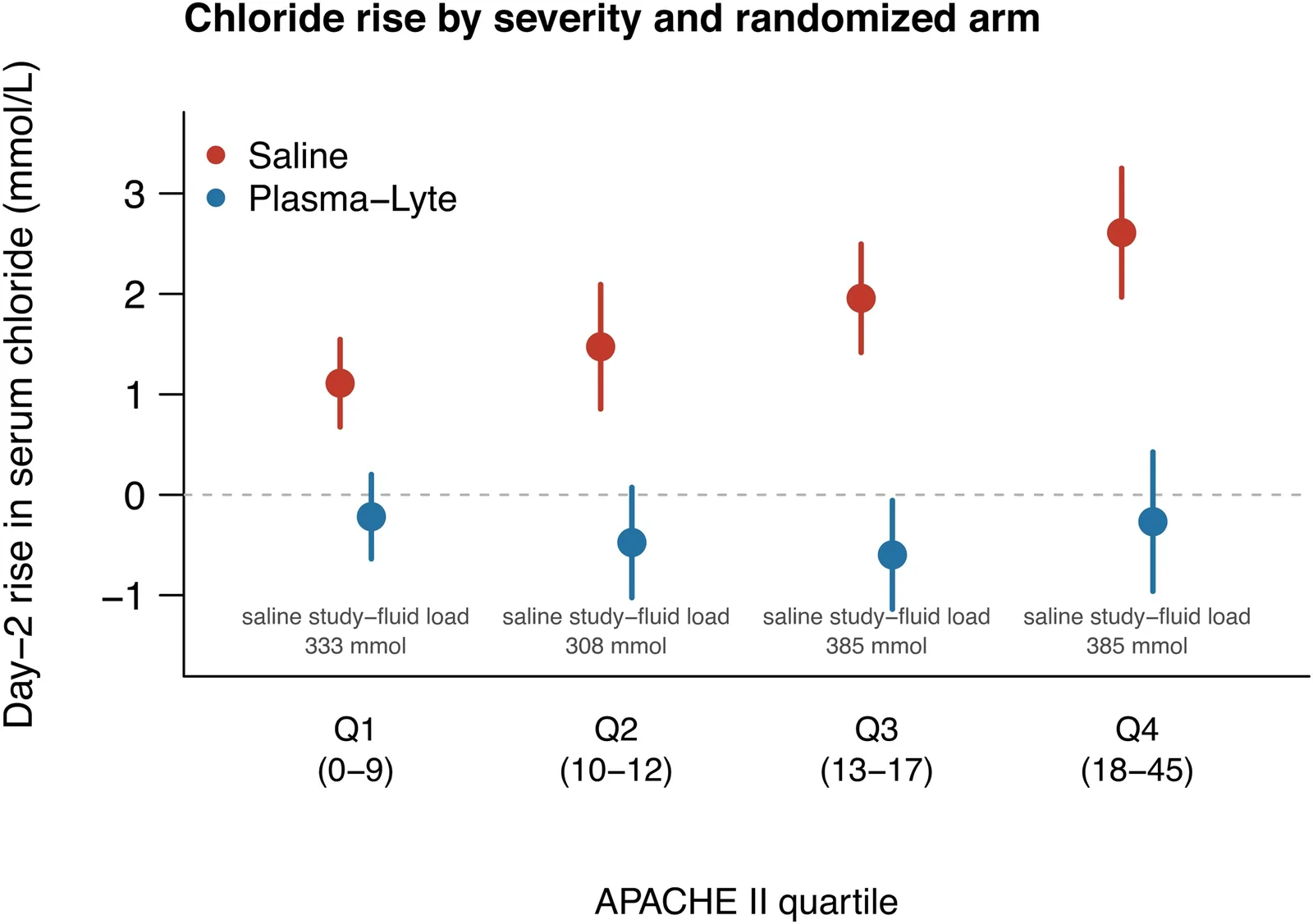
Severity and chloride rise. The analysis included patients with paired baseline and day-2 serum chloride measurements and was stratified by baseline APACHE II quartile. Day 2 was used because it balanced the accumulated between-group difference in study-fluid chloride load against attrition of paired measurements (see Methods). Points show mean change from baseline and vertical lines show 95% confidence intervals by randomized group. Annotations give median post-randomization study-fluid chloride load through day 2 among saline recipients only. Estimates are descriptive and unadjusted.

### Effect of randomized fluid by renal function

In the unadjusted descriptive trajectory, the saline-minus-Plasma-Lyte differences in mean change from baseline were +0.93 mmol/L (95% confidence interval, 0.66–1.20), +2.15 mmol/L (1.76–2.53), and +2.03 mmol/L (1.47–2.60) on days 1, 2, and 3, respectively (Fig. 2A). In the longitudinal model, the randomized between-group difference varied by baseline eGFR. For each 15 mL/min/1.73 m^2^ higher baseline eGFR, the randomized saline-versus-Plasma-Lyte difference was 0.29 mmol/L smaller (95% credible interval, 0.12 to 0.46 smaller) on day 2 and 0.34 mmol/L smaller (0.09 to 0.58 smaller) on day 3. The posterior probability of a negative arm-by-eGFR interaction was 99.96% on day 2 and 99.56% on day 3. At an eGFR of 30 mL/min/1.73 m², the between-group difference was +3.0 mmol/L (2.4–3.6) on day 2 and +3.0 mmol/L (2.2–3.9) on day 3. At an eGFR of 90 mL/min/1.73 m^2^, the corresponding differences were +1.8 mmol/L (1.5–2.2) and +1.7 mmol/L (1.1–2.2). The between-group difference was greater at lower baseline eGFR on both days 2 and 3. In the frequentist sensitivity models, the interaction was similar among 1,677 participants measured on all three days (day 2, 0.25 mmol/L smaller per 15 mL/min/1.73 m^2^ higher eGFR; 95% confidence interval, 0.03 to 0.47 smaller). When baseline creatinine replaced eGFR, the between-group difference was larger at higher creatinine, consistent in direction, but less precisely estimated (day 2, +0.33 mmol/L per standard deviation; 95% confidence interval, −0.03 to 0.69).

**Fig. 2:**
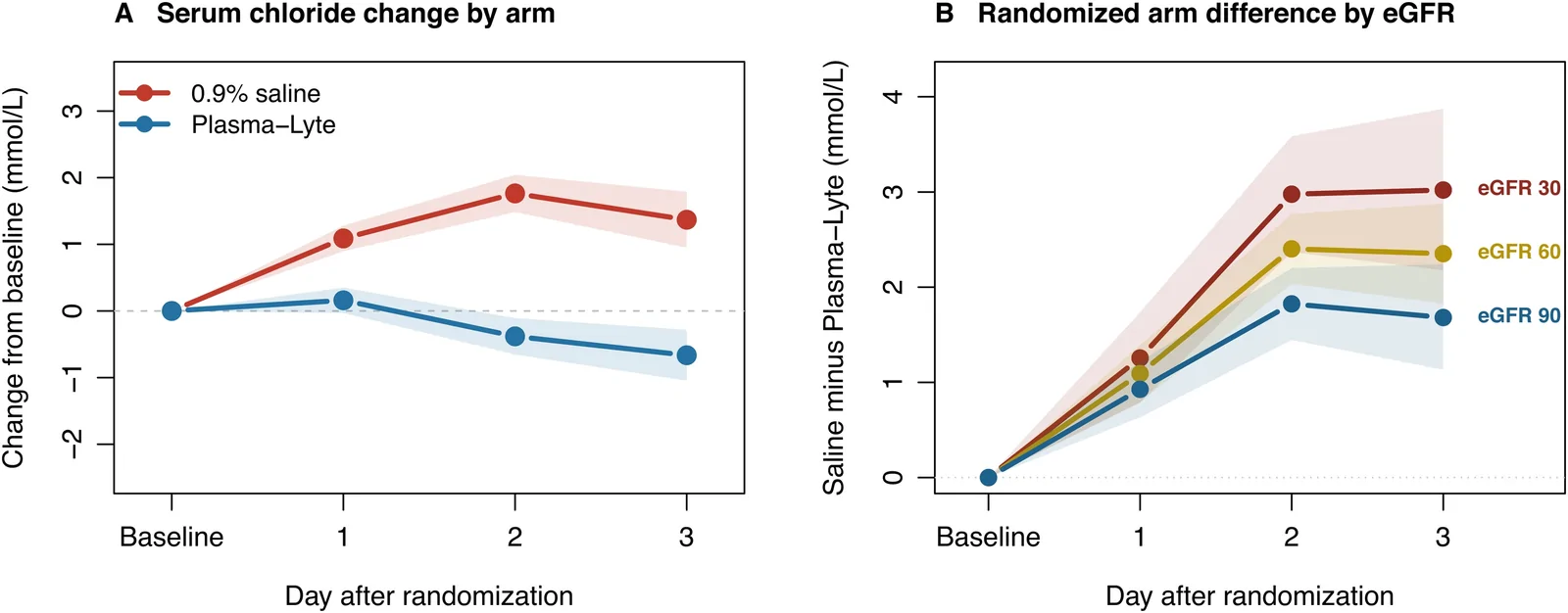
Serum chloride trajectory and randomized arm difference by renal function. (A) Mean change in serum chloride from baseline by randomized group from baseline (day 0, zero by definition) through day 3; shaded areas indicate 95% confidence intervals. (B) Estimated difference in serum chloride (saline minus Plasma-Lyte) by day at baseline eGFR values of 30, 60, and 90 mL/min/1.73 m²; day 0 is shown as the zero reference because the model conditions on baseline chloride. Points show posterior medians and shaded areas show 95% credible intervals. The difference was greater at lower baseline eGFR on days 2 and 3.

### Randomized normalization and within-saline association

Among 2,721 participants with paired day-2 data, serum chloride rose 2.15 mmol/L (95% confidence interval, 1.76–2.53) more with saline than with Plasma-Lyte, and saline recipients had received on average 145.7 mmol (127.0–164.4) more post-randomization study-fluid chloride. Dividing the first difference by the second gave a group-level ratio of +1.47 mmol/L per 100 mmol (bootstrap 95% confidence interval, 1.17–1.81). Among 1,321 complete-case saline recipients, the unweighted and inverse-probability-weighted observational associations were both +0.31 mmol/L per additional 100 mmol of study-fluid chloride (bootstrap 95% confidence intervals, 0.21−0.42 and 0.22−0.44; Fig. 3). Mean study-fluid volume differed by only 27 mL between groups, and the randomized differences implied an apparent distribution volume of 67.9 L (55.2–85.3). Missing adjustment-variable data, weighting diagnostics, and complete estimates are provided in Additional file 1 (Tables S1–S4).

**Fig. 3:**
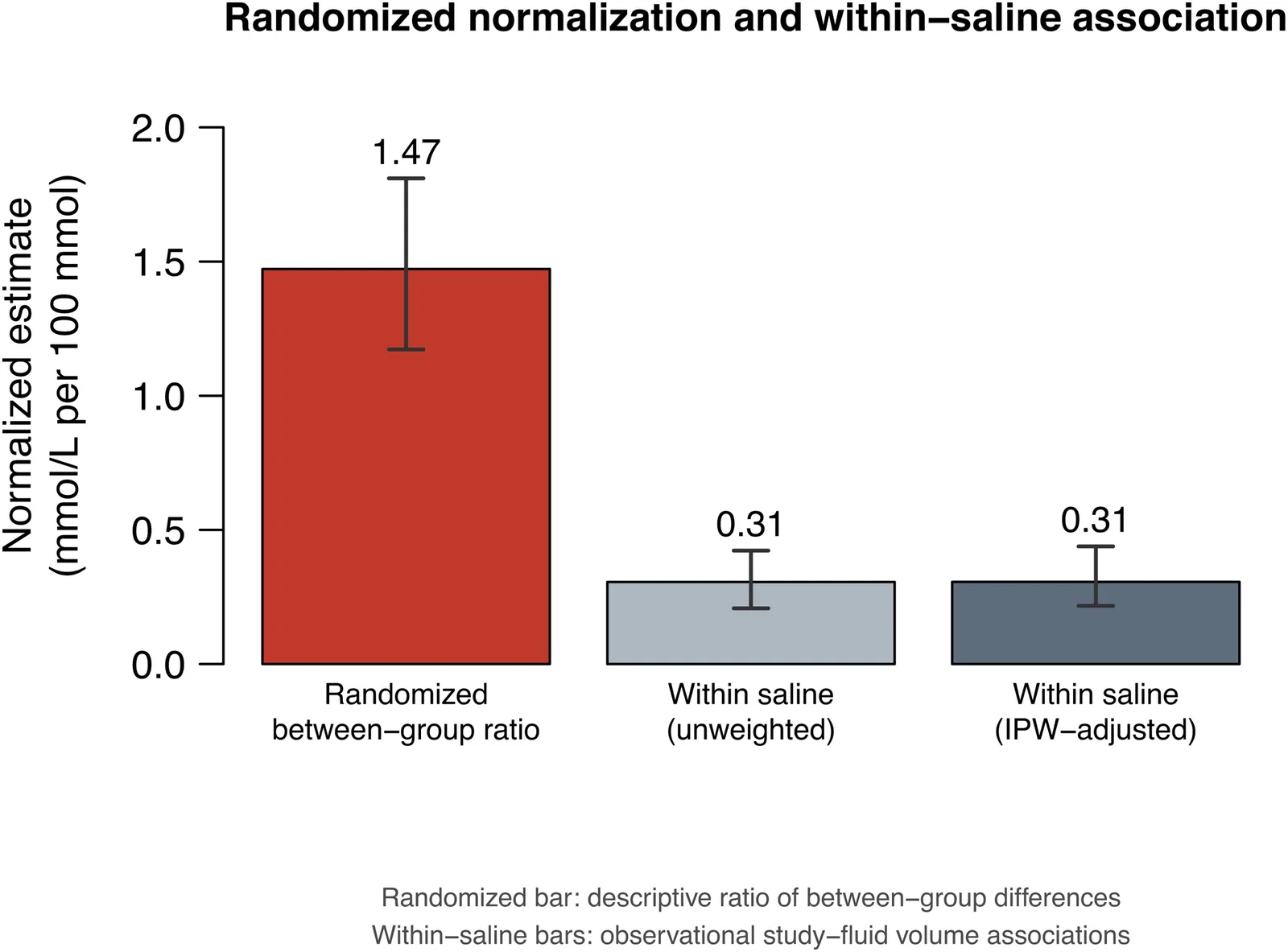
Randomized normalization and within-saline association. The first bar shows the group-level ratio of the randomized between-group difference in day-2 serum chloride change to the difference in post-randomization study-fluid chloride load, scaled per 100 mmol. The other bars show observational unweighted and inverse-probability-weighted associations per additional 100 mmol delivered as study saline. Lines show participant-bootstrap 95% confidence intervals.

### Variance partition

The analysis included 7,900 serum chloride measurements from 3,479 patients. The fluid component (study-fluid chloride load and total fluid volume) accounted for 0.7% (95% credible interval, 0.4–1.0) of modeled total variation in serum chloride and study day for 0.2% (0.1−0.3). Measured baseline characteristics accounted for 48.1% (46.3–49.8); baseline serum chloride alone accounted for 47.3% (45.4–49.0), and age, baseline creatinine, and SOFA score together for 0.4% (0.2−0.7). Shared variation among the correlated fixed predictors (covariance) was −0.3% (−0.5 to −0.1). Persistent between-patient differences accounted for 24.5% (22.9–26.1) and day-to-day within-patient fluctuation for 26.9% (25.6–28.2; Fig. 4); the corresponding standard deviations were 3.3 and 3.4 mmol/L. Restriction to 7,705 measurements from 3,453 patients with documented absence of kidney replacement therapy yielded a fluid component of 0.5% (0.3−0.8). With change from baseline as the outcome, the fluid component was 1.1% (0.7–1.7), with corresponding changes across the remaining components. With randomized arm added to the fluid component, the fluid component was 1.8% (1.3–2.4) and persistent between-patient differences 23.5% (22.0–25.1). In the exploratory analysis, all examined baseline variables and study site together explained 11% of the variation in estimated patient-specific intercepts. Higher baseline serum sodium and female sex were associated with modestly higher intercepts, and randomization to saline with a 0.8 mmol/L higher intercept; arterial pH, mean arterial pressure, vasopressor use, and admission type were not appreciably associated. Detailed methods and complete results are provided in Additional file 1 (Tables S5–S8).

**Fig. 4:**
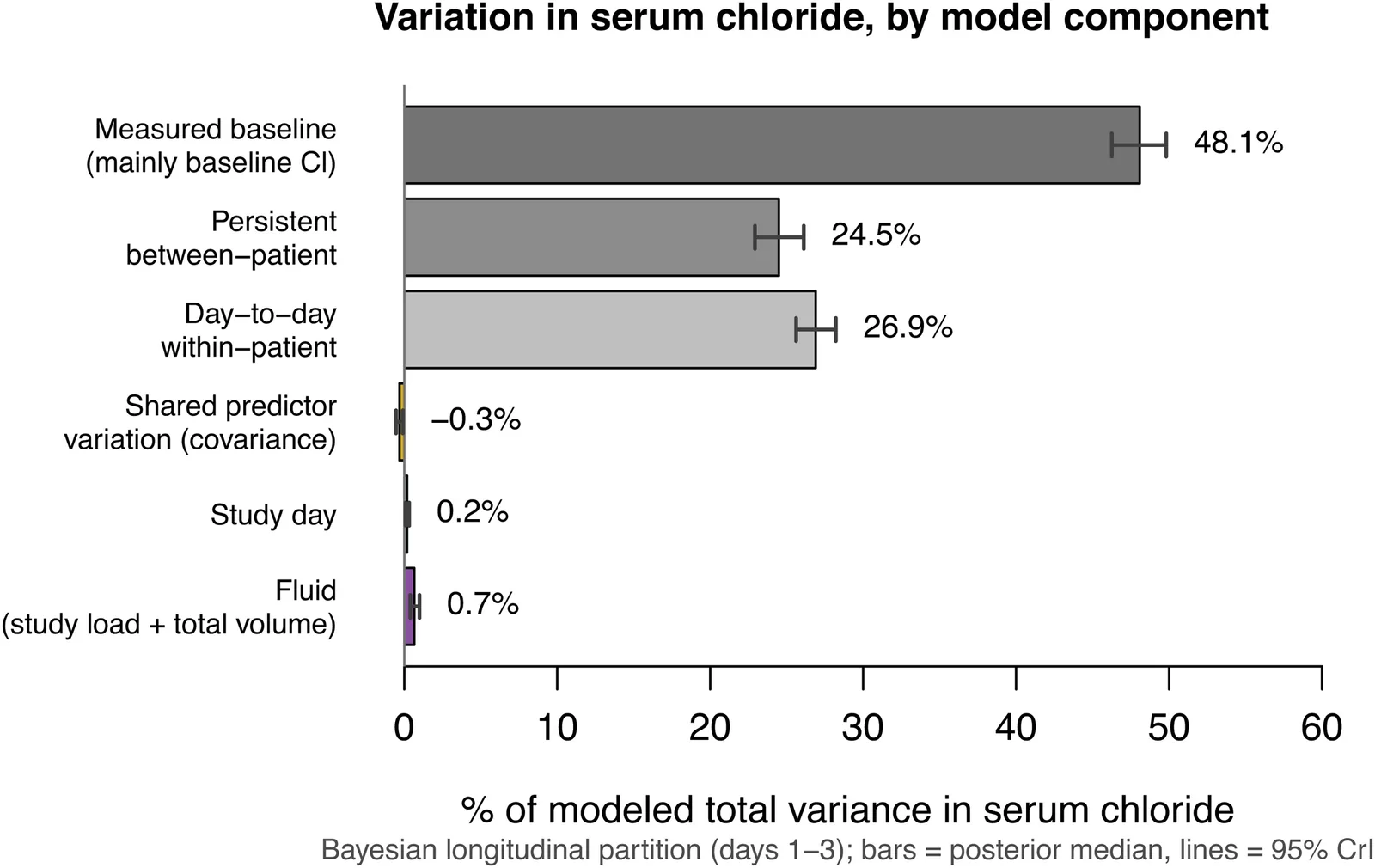
Variance partition. Bayesian partition of modeled total variation in observed serum chloride levels on days 1–3 into six additive components: fluid (study-fluid chloride load and total fluid volume); study day; measured baseline characteristics (baseline serum chloride, age, baseline creatinine, and SOFA score, of which baseline serum chloride accounted for nearly all); shared variation among these correlated predictors (covariance, which may be negative); persistent between-patient differences, i.e., each patient's consistent deviation from the values predicted by measured variables; and day-to-day fluctuation within patients. Bars show posterior medians and lines show 95% credible intervals.

### Non-study fluid sensitivity analysis

Total non-study-fluid volume through day 2 was small (median 120 mL with saline and 200 mL with Plasma-Lyte), and the mean between-group difference was −4.3 mL. The study-fluid chloride-load gap was +145.7 mmol; conservatively pricing the mean non-study-volume difference at either 98 or 154 mmol/L yielded gaps of +145.3 and +145.0 mmol, respectively.

## Discussion

In this analysis of the BaSICS trial, randomization to saline produced an approximately 2 mmol/L greater increase in serum chloride than Plasma-Lyte by day 2. The randomized between-group difference was greater at lower baseline eGFR on days 2 and 3. In the descriptive variance model, the fluid component was small relative to baseline serum chloride and to persistent patient-specific differences.

SPLIT [8], SALT-ED [9], SMART [2], BaSICS [3], and PLUS [4] consistently demonstrated lower serum chloride with balanced crystalloids and neutral or near-neutral mortality results. An individual-patient meta-analysis suggested at most a small mortality benefit [5], and the more recent FLUID cluster-crossover trial in hospitalized patients reported similar rates of death or hospital readmission [10]. The present analysis focuses on the early serum chloride response. A secondary analysis of PLUS estimated similar randomized-fluid effects on 90-day mortality across quartiles of baseline serum chloride and pH [11]. Its estimand concerned effect modification by baseline values. Our observed separation of approximately 2 mmol/L quantifies the early biochemical response; clinical toxicity thresholds require different designs and broader exposure ranges.

The variance-partition analysis quantified statistical variation in observed serum chloride levels. After inclusion of study day, the fluid component accounted for 0.7% of modeled total variation. Nearly half was explained by measured baseline characteristics, almost entirely by baseline serum chloride: patients who started with higher chloride tended to remain higher. About one quarter reflected persistent between-patient differences, and a similar share reflected day-to-day fluctuation within patients. A small share of variation does not make the randomized effect negligible: a 2 mmol/L shift applied to a whole population is small relative to the spread of chloride values between critically ill patients, yet it is consistent and reproducible. Restriction to measurements without kidney replacement therapy yielded a similarly small fluid component. With change from baseline as the outcome, the fluid component was 1.1%, with corresponding reallocation across the remaining components.

Persistent between-patient differences are, by construction, variation that measured characteristics did not capture. Serum chloride reflects free-water balance, electroneutrality with accompanying ions, and renal handling relative to sodium [[12], [13], [14]]. Consistent with this, higher baseline serum sodium was associated with higher patient-specific intercepts. Randomization to saline was also associated with a higher intercept, indicating that part of the randomized fluid effect not captured by the cumulative load term appeared in this component; adding randomized arm to the fluid component raised that component from 0.7% to 1.8%, which remained small. However, measured baseline variables and study site together explained only about one tenth of this component. The remainder probably reflects unmeasured determinants, such as free-water gains and losses, acid-base status, renal chloride handling, and differences in laboratory methods.

A principal strength of this study is randomized assignment, which supports estimation of the effect of assigned fluid type on serum chloride. Saline and Plasma-Lyte differ in chloride and other constituents, making assigned fluid type the randomized estimand. The analyses characterize the early biochemical response to randomized fluid strategies. Assessment of chloride mediation would require longitudinal mediator and clinical-outcome data.

These findings suggest several possible, though speculative, interpretations of the small clinical effects of balanced crystalloids. First, because fluid type shifted serum chloride by only about 2 mmol/L, crystalloid choice may have little clinically important effect on kidney function or mortality through chloride. Second, if balanced crystalloids do have modest benefits, these may be mediated mainly by mechanisms other than chloride, such as acid-base effects or other constituents; our design cannot separate these because the fluids differ in multiple constituents. Third, if chloride remains an important mediator, as experimental studies of renal perfusion suggest [7], baseline chloride and patient-specific factors, which dominated chloride variation, may matter more than crystalloid type. Any benefit of balanced solutions would then be concentrated in subgroups with larger chloride separation, such as patients with reduced renal function, in whom the between-group difference was about 3 mmol/L, or those receiving large volumes. Similar mortality effects across baseline chloride quartiles in PLUS [11] argue against strong effect modification by baseline chloride. Distinguishing these possibilities would require mediation analyses with longitudinal chloride and clinical-outcome data, or trials enriched for patients expected to have the largest chloride separation.

This analysis has limitations. First, serum chloride was measured at clinicians' discretion in approximately one third of participants, limiting generalizability and potentially introducing informative sampling. Second, the randomized fluids differed in multiple constituents, making assigned fluid type the causal estimand. Third, the primary exposure included only post-randomization study fluid. Non-study-fluid composition was unavailable; sensitivity analyses based on total non-study volume produced similar day-2 load gaps. Fourth, follow-up covered the first three days after randomization and therefore characterized the early chloride response rather than trajectories over the entire ICU stay. Fifth, kidney replacement therapy could alter serum chloride; restriction to documented absence of therapy yielded a similar fluid component. Direct measurement of chloride removal was unavailable. Sixth, 53.5% of the analytic cohort was admitted after planned surgery, centering generalizability on similar ICU populations. Finally, urinary chloride and serial acid-base measurements, which could help explain persistent between-patient differences, were unavailable.

## Conclusion

Over the first three ICU days, randomization to saline increased serum chloride relative to Plasma-Lyte, with a greater difference between randomized groups at lower baseline eGFR on days 2 and 3. In a descriptive variance model, the fluid component was small relative to baseline serum chloride and to persistent patient-specific differences that measured characteristics did not explain.

## Authors’ contributions

FGZ conceived the study, performed the analysis, and drafted the manuscript. ISM, FRM, and ABC contributed to the conduct of the BaSICS trial and to funding acquisition, and critically revised the manuscript for important intellectual content. All authors read and approved the final manuscript.

## Consent for publication

Not applicable.

## Ethics approval and consent to participate

The BaSICS trial was approved by the Brazilian National Research Ethics Commission (CONEP) and by the research ethics boards of all participating centers, and was conducted in accordance with the Declaration of Helsinki; written informed consent was obtained from participants or their legal surrogates in accordance with the trial protocol (ClinicalTrials.gov, NCT02875873). The present work is a secondary analysis of fully anonymized data and, in accordance with local guidelines, required no additional ethics approval or participant consent.

## Declaration of Generative AI and AI-assisted technologies in the writing process

During the preparation of this work the authors used large language models (Claude, Anthropic, and Codex, OpenAI) to assist with creating scripts and checking analysis, as well as editing text for clarity. All analyses, results, and interpretations were verified by the authors, who reviewed and edited the content as needed and take full responsibility for the publication’s content. Grammarly was used for further spell checking and improving reading flow.

## Funding

This secondary analysis received no specific funding. The BaSICS trial was funded by the Brazilian Ministry of Health.

## Availability of data and material

The BaSICS dataset is not publicly available. It is governed by the BaSICS/BRICNet data-sharing policy and may be made available on reasonable request, subject to appropriate ethics and data-transfer approvals. The analysis code is openly available at https://github.com/fzampier/basics-chloride-analysis and archived at Zenodo (https://doi.org/10.5281/zenodo.23025078).

## Declaration of competing interest

The authors declare that they have no competing interests
