# Supplementary material for: Chloride load and serum chloride in critically ill adults: a randomization-anchored analysis of the BaSICS trial
Source: Ann Intensive Care. 2026 Oct 22;16:100191. doi: 10.1016/j.aicoj.2026.100191 (PMC13742502; doi:10.1016/j.aicoj.2026.100191)
Supplement: Supplementary file 1 [file mmc1.pdf]

Additional file 1

Supplementary statistical methods, diagnostics, and sensitivity analyses

Related manuscript: *Chloride load and serum chloride in critically ill adults: a randomization-anchored analysis of the BaSICS trial*

This file provides additional methods and diagnostics for the observational within-saline generalized propensity-score analysis and the Bayesian partition of modeled variation in serum chloride.

1. Observational within-saline analysis

1.1 Analysis cohort, exposure, and outcome

This analysis included participants randomized to saline who had baseline and day-2 serum chloride measurements, cumulative post-randomization study-fluid chloride load through day 2, and complete data for the adjustment variables. Of 1,357 saline recipients with paired baseline and day-2 chloride measurements and a study-fluid chloride-load value, 1,321 were complete cases for the adjustment set.

The exposure was cumulative post-randomization study-fluid chloride load through day 2, expressed in mmol. Because all participants in this analysis received study saline at a fixed chloride concentration of 154 mmol/L, variation in study-fluid chloride load represented variation in clinician-determined study-saline volume. The outcome was change in serum chloride from baseline to day 2, expressed in mmol/L.

Table S1. Missing adjustment variables among 1,357 paired saline recipients

Variable	Missing, No.
Baseline creatinine	0
Body weight	36
SOFA score	1
Mean arterial pressure	1
Age	0
Baseline serum chloride	0
Missing at least one adjustment variable	36

Missingness in individual variables overlapped. No missing values were imputed.

1.2 Generalized propensity-score model and stabilized weights

We estimated a generalized propensity score for the continuous exposure using the following Gaussian linear model among the 1,321 complete-case saline recipients:

$$A_i = \gamma_0 + \gamma_1 \text{Creatinine}_i + \gamma_2 \text{Weight}_i + \gamma_3 \text{SOFA}_i + \gamma_4 \text{MAP}_i + \gamma_5 \text{Age}_i + \gamma_6 \text{Chloride}_{0i} + \varepsilon_i,$$

where A_i was cumulative study-fluid chloride load through day 2 and ε_i followed a normal distribution with constant variance. The adjustment variables were baseline creatinine, measured body weight, SOFA score, mean arterial pressure, age, and baseline serum chloride.

The stabilized weight for participant i was

$$SW_i = \frac{f_A(A_i)}{f_{A|X}(A_i \mid X_i)},$$

where the numerator was the normal density evaluated using the observed marginal mean and standard deviation of chloride load and the denominator was the normal density evaluated using the participant-specific fitted mean and residual standard deviation from the generalized propensity-score model. We capped weights above the sample 99th percentile at the 99th-percentile value. The weights were not otherwise rescaled.

In the complete-case cohort, mean cumulative study-fluid chloride load was 406.7 mmol (standard deviation, 287.3; median, 351.4; range, 0-4,157.4). The generalized propensity-score model had an R^2 of 0.015 and a residual standard deviation of 285.8 mmol.

1.3 Outcome models and uncertainty

We fitted unweighted and weighted Gaussian linear models with day-2 change in serum chloride as the outcome and cumulative study-fluid chloride load as a continuous predictor:

$$\Delta\text{Chloride}_{2i} = \alpha_0 + \alpha_1 A_i + e_i.$$

The weighted model used the stabilized generalized propensity-score weights. Coefficients were multiplied by 100 and are reported as the mean difference in day-2 serum chloride change per additional 100 mmol of study-fluid chloride delivered as saline.

We calculated 95% confidence intervals using a nonparametric participant bootstrap with 10,000 replicates and random seed 20240608. Within each bootstrap sample, we refitted the generalized propensity-score model, recalculated and capped the weights using the bootstrap-sample 99th percentile, and refitted both outcome models. Confidence limits were the 2.5th and 97.5th percentiles of the finite bootstrap estimates.

1.4 Weight and balance diagnostics

Table S2. Stabilized-weight diagnostics

Diagnostic	Before capping	After 99th-percentile capping
Mean	1.000	0.996
Standard deviation	0.127	0.108
Minimum	0.294	0.294
Median	0.995	0.995
95th percentile	1.160	1.160
99th percentile	1.433	1.432
Maximum	2.234	1.433
Effective sample size, No.	1,300	1,306

Effective sample size was calculated as $(\sum_i SW_i)^2 / \sum_i SW_i^2$.

For a continuous exposure, balance was assessed using the Pearson correlation between chloride load and each baseline adjustment variable before weighting and the corresponding weighted correlation after weighting. Table S3 reports signed correlations; the largest absolute correlation after 99th-percentile capping was 0.023.

Table S3. Correlation between study-fluid chloride load and adjustment variables

Variable	Unweighted	Weighted before capping	Weighted after capping
Baseline creatinine	0.021	-0.016	-0.007
Body weight	0.016	0.027	0.014
SOFA score	-0.005	-0.006	-0.006

Variable	Unweighted	Weighted before capping	Weighted after capping
Mean arterial pressure	0.059	-0.003	0.010
Age	0.098	0.019	0.023
Baseline serum chloride	0.032	-0.026	-0.014

1.5 Within-saline estimates

Table S4. Observational association between study-fluid chloride load and day-2 serum chloride change

Model	Participants, No.	Estimate per additional 100 mmol, mmol/L	Bootstrap 95% CI
Unweighted	1,321	0.31	0.21 to 0.42
Generalized propensity-score weighted	1,321	0.31	0.22 to 0.44

2. Bayesian partition of modeled variation in serum chloride

2.1 Analysis cohort and model specification

The primary analysis included 7,900 serum chloride measurements from 3,479 participants. The outcome was the observed serum chloride value on study days 1-3. Continuous predictors were standardized to a mean of 0 and standard deviation of 1 within the analytic dataset. Study day was represented by indicator variables for days 2 and 3, with day 1 as the reference.

For participant i on study day j, the Gaussian hierarchical model was

$$Y_{ij} = \beta_0 + X_{F,ij}\beta_F + X_{D,ij}\beta_D + X_{P,i}\beta_P + u_i + \epsilon_{ij},$$

where:

- Y_{ij} was the observed serum chloride value;
- X_F contained cumulative study-fluid chloride load and cumulative total fluid volume;
- X_D contained the day-2 and day-3 indicators;
- X_P contained baseline chloride, age, baseline creatinine, and baseline SOFA score;
- $u_i \sim N(0, \sigma_u^2)$ was a participant-specific random intercept; and
- $\epsilon_{ij} \sim N(0, \sigma_e^2)$ was the within-participant residual.

2.2 Priors and computation

Fixed-effect slopes had normal(0, 5) priors in all three models. In the primary serum-chloride-level model and the kidney-replacement-therapy-restricted model, the intercept had a Student-t(3, 106, 4.4) prior, and the participant-level and residual standard deviations had half-Student-t(3, 0, 4.4) priors. In the change-from-baseline sensitivity model, the intercept had a Student-t(3, 0, 3) prior, and the participant-level and residual standard deviations had half-Student-t(3, 0, 3) priors. Models were fitted in R 4.6.1 using brms 2.23.0 with the rstan 2.32.7 backend.

Each model used four chains of 4,000 iterations, including 2,000 warm-up iterations per chain. The primary model used random seed 1; the kidney-replacement-therapy-restricted and change-from-baseline sensitivity models used seeds 16017 and 17017, respectively. Convergence assessment included R-hat, bulk and tail effective sample sizes, and divergent transitions.

2.3 Partition of modeled total variation

For each posterior draw, we grouped the fixed predictors into fluid, study-day, and measured baseline-characteristic blocks. Let Σ_F, Σ_D, and Σ_P denote the observed covariance matrices of the predictors within these blocks. Their variation components were

$$V_F = \beta_F^T \Sigma_F \beta_F, \qquad V_D = \beta_D^T \Sigma_D \beta_D, \qquad V_P = \beta_P^T \Sigma_P \beta_P.$$

Let X_{all} contain all fixed predictors and Σ_{all} denote their joint covariance matrix. The total fixed-predictor variation was

$$V_{fixed} = \beta_{all}^T \Sigma_{all} \beta_{all}.$$

The covariance component among the fixed-predictor blocks was

$$V_{cov} = V_{fixed} - V_F - V_D - V_P.$$

This component retains the cross-covariances generated by correlations among the fluid, study-day, and baseline-characteristic predictor blocks and can therefore be negative. The between-participant and within-participant components were

$$V_B = \sigma_u^2, \qquad V_R = \sigma_e^2.$$

Modeled total variation was

$$V_{total} = V_{fixed} + V_B + V_R.$$

For each posterior draw, the relative contribution of component k was calculated as $100 \times V_k/V_{total}$. We summarized each posterior distribution using its median and 2.5th and 97.5th percentiles.

2.4 Sensitivity analyses

The kidney-replacement-therapy sensitivity analysis excluded measurements obtained during or after documented kidney replacement therapy through the measurement day and measurements with unknown daily kidney-replacement-therapy status. Of the 7,900 primary-analysis measurements, 193 measurements from 129 participants were excluded because of kidney replacement therapy and 2 measurements from 1 participant because daily status was unknown. The restricted analysis included 7,705 measurements from 3,453 participants.

The change-from-baseline sensitivity analysis used change in serum chloride from baseline as the outcome and otherwise retained the primary model structure and analytic observations. Continuous predictors were restandardized within each sensitivity-analysis dataset.

The randomized-arm sensitivity analysis added an indicator for randomization to saline to the fluid block of the primary model, so that the fluid component comprised randomized arm, cumulative study-fluid chloride load, and cumulative total fluid volume. It otherwise retained the primary model structure, priors, sampler settings, and analytic observations. This analysis was added in the second revision after the exploratory analysis in Section 2.8 showed that randomization to saline was associated with higher patient-specific intercepts, indicating that part of the randomized fluid effect not captured by the chloride-load term had been absorbed into the between-participant component.

2.5 Relative-contribution results

Table S5. Bayesian partition of modeled total variation

Analysis	Component	Posterior median, %	95% credible interval, %
Primary serum chloride level	Fluid	0.65	0.39 to 0.99
	Study day	0.18	0.09 to 0.32
	Measured baseline characteristics	48.08	46.26 to 49.81
	Shared predictor variation (covariance)	-0.31	-0.54 to -0.09
	Persistent between-participant differences	24.49	22.92 to 26.12
	Day-to-day within-participant fluctuation	26.89	25.62 to 28.21
Excluding KRT-exposed and unknown-status measurements	Fluid	0.54	0.29 to 0.85
	Study day	0.11	0.04 to 0.22
	Measured baseline characteristics	48.99	47.19 to 50.70
	Shared predictor variation (covariance)	-0.27	-0.51 to -0.03
	Persistent between-participant differences	24.49	22.94 to 26.16
	Day-to-day within-participant fluctuation	26.11	24.86 to 27.45
Change from baseline outcome	Fluid	1.11	0.66 to 1.66
	Study day	0.31	0.14 to 0.55
	Measured baseline characteristics	12.57	10.90 to 14.29
	Shared predictor variation (covariance)	-0.15	-0.47 to 0.13
	Persistent between-participant differences	41.08	38.81 to 43.41
	Day-to-day within-participant fluctuation	45.03	42.93 to 47.16
Randomized arm added to fluid component	Fluid (including randomized arm)	1.79	1.31 to 2.35
	Study day	0.19	0.08 to 0.33
	Measured baseline characteristics	47.30	45.58 to 49.04
	Shared predictor variation (covariance)	0.35	0.10 to 0.60
	Persistent between-participant differences	23.49	21.96 to 25.09
	Day-to-day within-participant fluctuation	26.84	25.59 to 28.17

KRT = kidney replacement therapy.

2.6 Bayesian model diagnostics

Table S6. Sampling diagnostics

Analysis	Maximum R-hat	Minimum bulk effective sample size	Minimum tail effective sample size	Divergent transitions, No.
Primary serum chloride level	1.002	2,571	4,234	0
Excluding KRT-exposed and unknown-status measurements	1.001	2,535	4,030	0
Change from baseline outcome	1.001	2,681	4,209	0
Randomized arm added to fluid component	1.002	2,291	4,447	0

2.7 Decomposition of the measured-baseline component

To identify which measured characteristics contributed to the measured-baseline component of the primary model, we calculated, for each posterior draw, the variation attributable to baseline serum chloride alone and to age, baseline creatinine, and SOFA score together, using the same predictor-covariance approach as in Section 2.3. The remainder of the component is the covariance between these two subsets.

Table S7. Decomposition of the measured-baseline component (primary serum chloride level model)

Quantity	Posterior median	95% credible interval
Measured baseline characteristics, % of modeled total variation	48.08	46.26 to 49.81
Baseline serum chloride alone, %	47.25	45.38 to 48.96
Age, baseline creatinine, and SOFA score together, %	0.43	0.21 to 0.73
Covariance between these subsets, %	0.41	0.13 to 0.66
Baseline serum chloride, % of the measured-baseline component	98.27	97.25 to 99.13
Between-participant standard deviation, mmol/L	3.26	3.14 to 3.38
Within-participant residual standard deviation, mmol/L	3.42	3.35 to 3.49

2.8 Exploratory correlates of persistent between-participant differences

We extracted the posterior mean of each participant's random intercept from the primary model (3,479 participants) and regressed these estimates on baseline variables not included in the variance model: serum sodium, arterial pH, mean arterial pressure, measured body weight, pre-enrollment saline volume (24 hours before randomization), APACHE II score, vasopressor use, sex, admission type, and randomized arm. We then added study site (53 sites) as a categorical variable. Continuous variables were standardized. The analysis used 3,081 participants with complete data. Because the intercepts are shrunken posterior estimates and the analysis was not prespecified, the results are descriptive and hypothesis-generating.

Table S8. Associations of baseline variables with estimated patient-specific intercepts (mmol/L)

Variable	Estimate	95% confidence interval	P value
Serum sodium, per SD	0.21	0.12 to 0.30	<0.001
Arterial pH, per SD	-0.01	-0.11 to 0.09	0.78
Mean arterial pressure, per SD	-0.06	-0.15 to 0.04	0.24
Measured body weight, per SD	0.10	0.00 to 0.19	0.04
Pre-enrollment saline volume, per SD	-0.12	-0.21 to -0.03	0.007
APACHE II score, per SD	0.07	-0.04 to 0.19	0.20
Randomized to saline	0.85	0.67 to 1.04	<0.001
Female sex	0.42	0.22 to 0.61	<0.001
Vasopressor use at randomization	-0.06	-0.25 to 0.14	0.56
Admission: unplanned, non-sepsis (vs elective)	-0.16	-0.40 to 0.08	0.20
Admission: unplanned, sepsis (vs elective)	-0.04	-0.35 to 0.27	0.79

Variance of the estimated intercepts explained (R^2): baseline variables, 4.4%; study site alone, 6.4%; baseline variables and study site together, 10.7%. SD = standard deviation.
